# Supplementary figures and images for: The Rpd3 histone deacetylase is a critical regulator of temperature-mediated morphogenesis and virulence in the human fungal pathogen Histoplasma
Source: PLoS Biol. 2026 Mar 17;24(3):e3003341. doi: 10.1371/journal.pbio.3003341 (PMC13132440; doi:10.1371/journal.pbio.3003341)

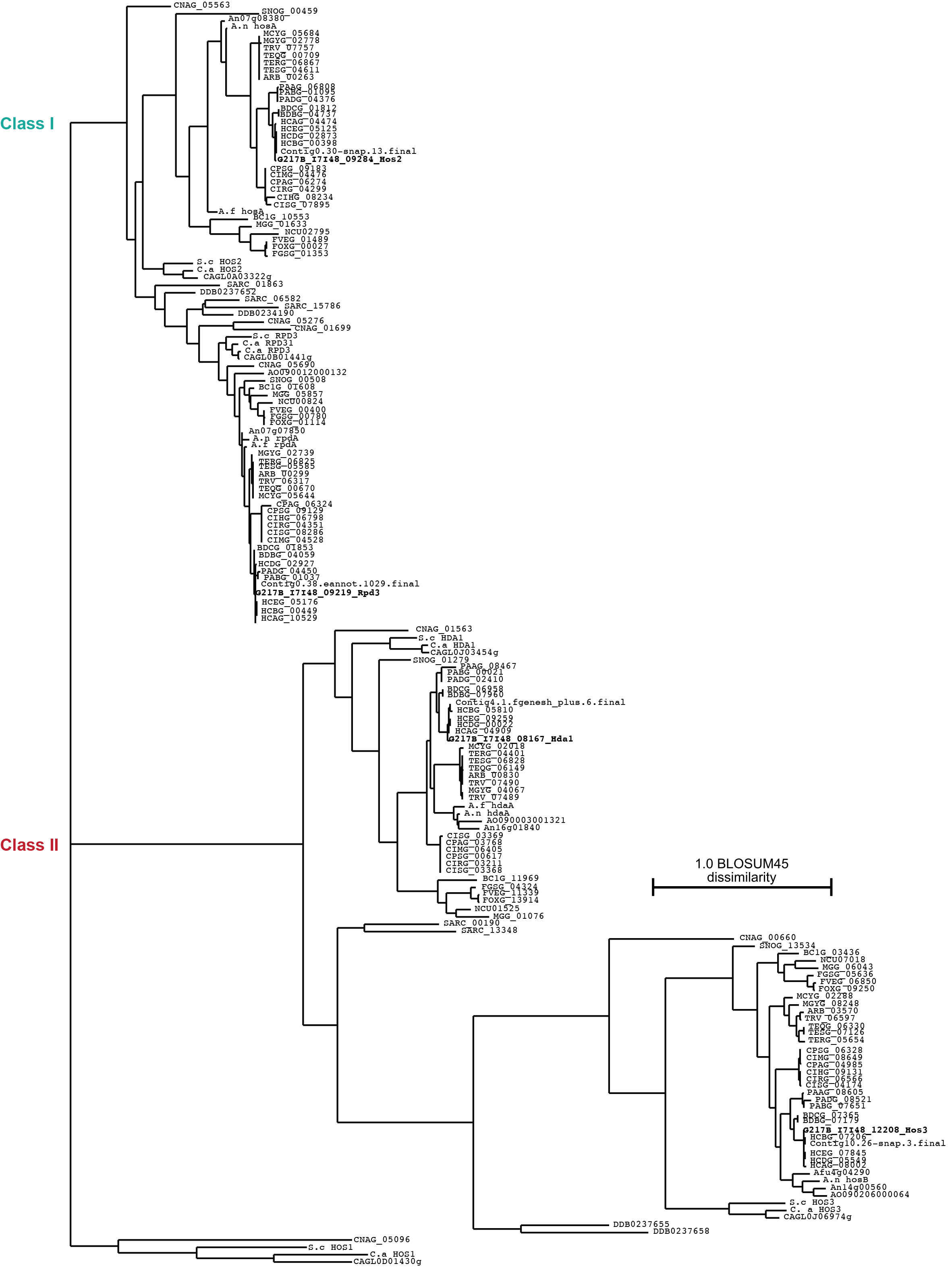

Supplement: S1 Fig — Phylogenetic tree of classic HDAC-encoding genes in a panel of 41 fungal proteomes encompassing human fungal pathogens as well as environmental, entomopathogenic, and phytopathogenic fungi. S5 Table contains the full list of gene names and their corresponding genomes. Genes found in the G217B laboratory strain used in this study are bolded. The underlying tree file can be found in S2 Data. (PNG) [file pbio.3003341.s001.png]

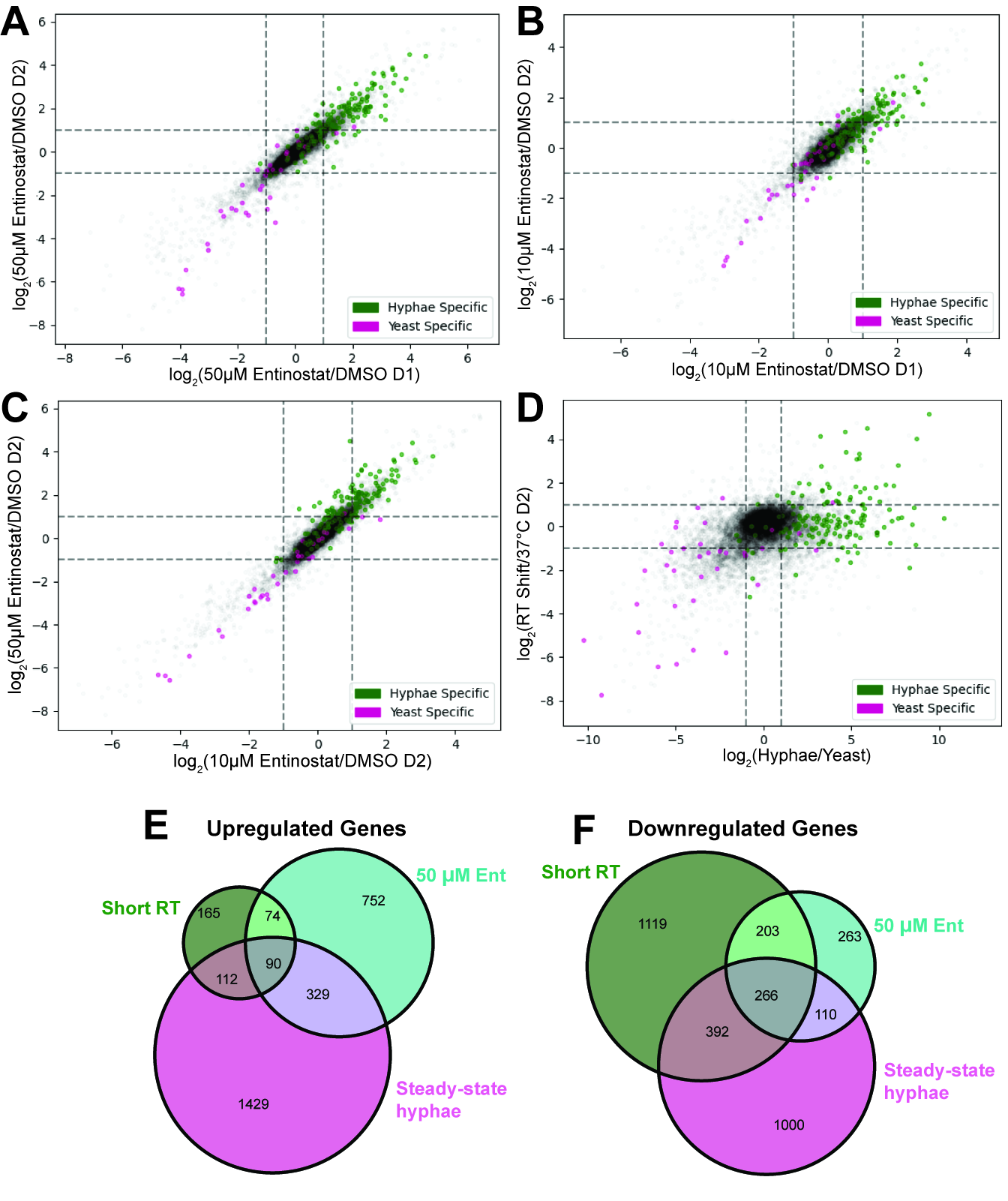

Supplement: S2 Fig — Scatterplots comparing differential RNA-seq signal in all G217B transcripts between 50 µM of Entinostat on day 1 and day 2 (A), 10 µM of Entinostat on day 1 and day 2 (B), 10 and 50 µM of Entinostat on day 2 (C), and 2-day RT shift and steady-state hyphae (D) (Gilmore and colleagues [24]). Venn diagrams comparing the regulons of 2-day RT (short RT), 50 µM Entinostat, and steady-state hyphae (previously published in Gilmore and colleagues [24]) to identify shared and unique upregulated (E) and downregulated genes (F). Underlying data can be found in S6 Table. (TIF) [file pbio.3003341.s002.tif]

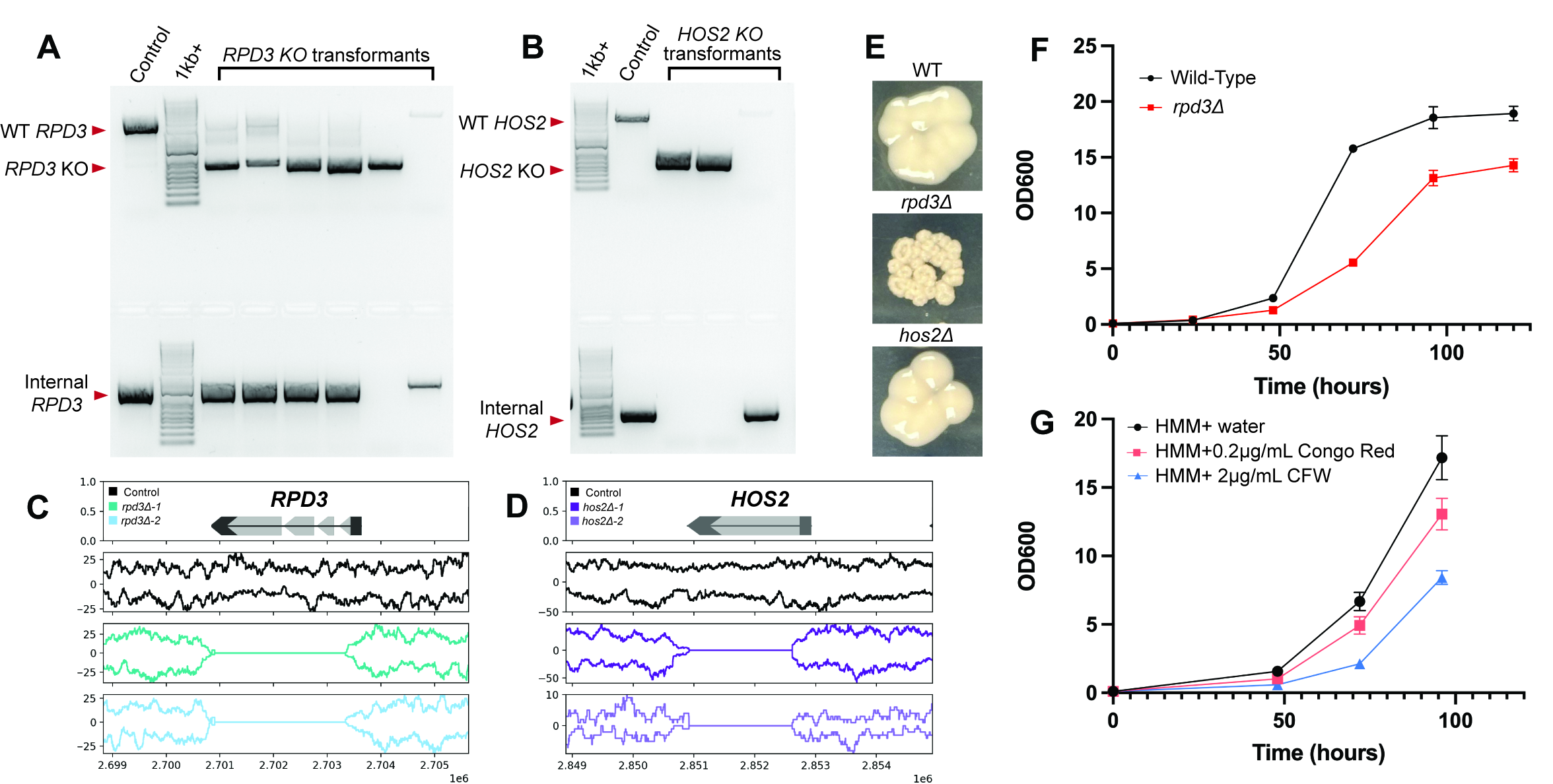

Supplement: S3 Fig — Colony PCR products run on a 1% Agarose gel to identify successful KO isolates for RPD3 (A) and HOS2 (B). The top half of each gel contains PCR products amplified with a primer pair external to the sgRNA sites that is indicated in Fig 3A. The bottom half uses a primer pair that flanks both sides of the 5′ sgRNA cut site such that negative signal is indicative of clean KO. Coverage tracks displaying sequencing signal at the chromosomal coordinates surrounding RPD3 for control and rpd3Δ strains (C), and at HOS2 for control and hos2Δ strains (D). The underlying data can be found in SRA SRP593199. E. Plate morphology phenotypes of WT, rpd3Δ, and hos2Δ strains. F. OD600 growth curve of WT and rpd3Δ strains grown for 5 days at 37 °C. Points represent the average OD600 values of samples in triplicate. G. OD600 growth curve of rpd3Δ passaged at 37 °C for 4 days in the presence of cell wall stressors. Points represent the average OD600 values of samples in triplicate. Underlying data can be found in S16 Table. (TIF) [file pbio.3003341.s003.tif]

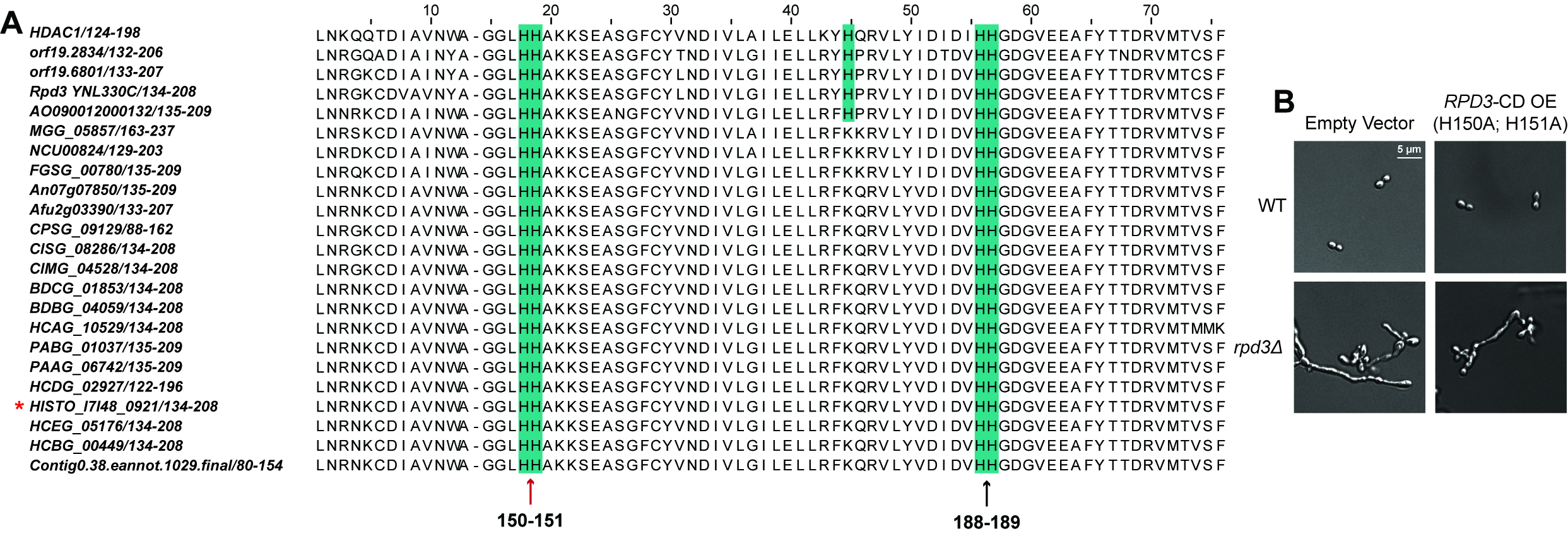

Supplement: S4 Fig — A. Alignment of the Rpd3 HDAC domain across fungal species and alongside human HDAC1 (HDAC1/124-198). Histoplasma G217B Rpd3 is indicated by the red asterisk. The red arrows indicate the 150H and 151H residues observed to be critical for catalytic activity in orthologous species. B. Representative micrographs of transformants yielded following transformation of WT and rpd3Δ strains with an empty vector or a plasmid overexpressing a Catalytic Dead (CD) variant of Rpd3. (TIF) [file pbio.3003341.s004.tif]

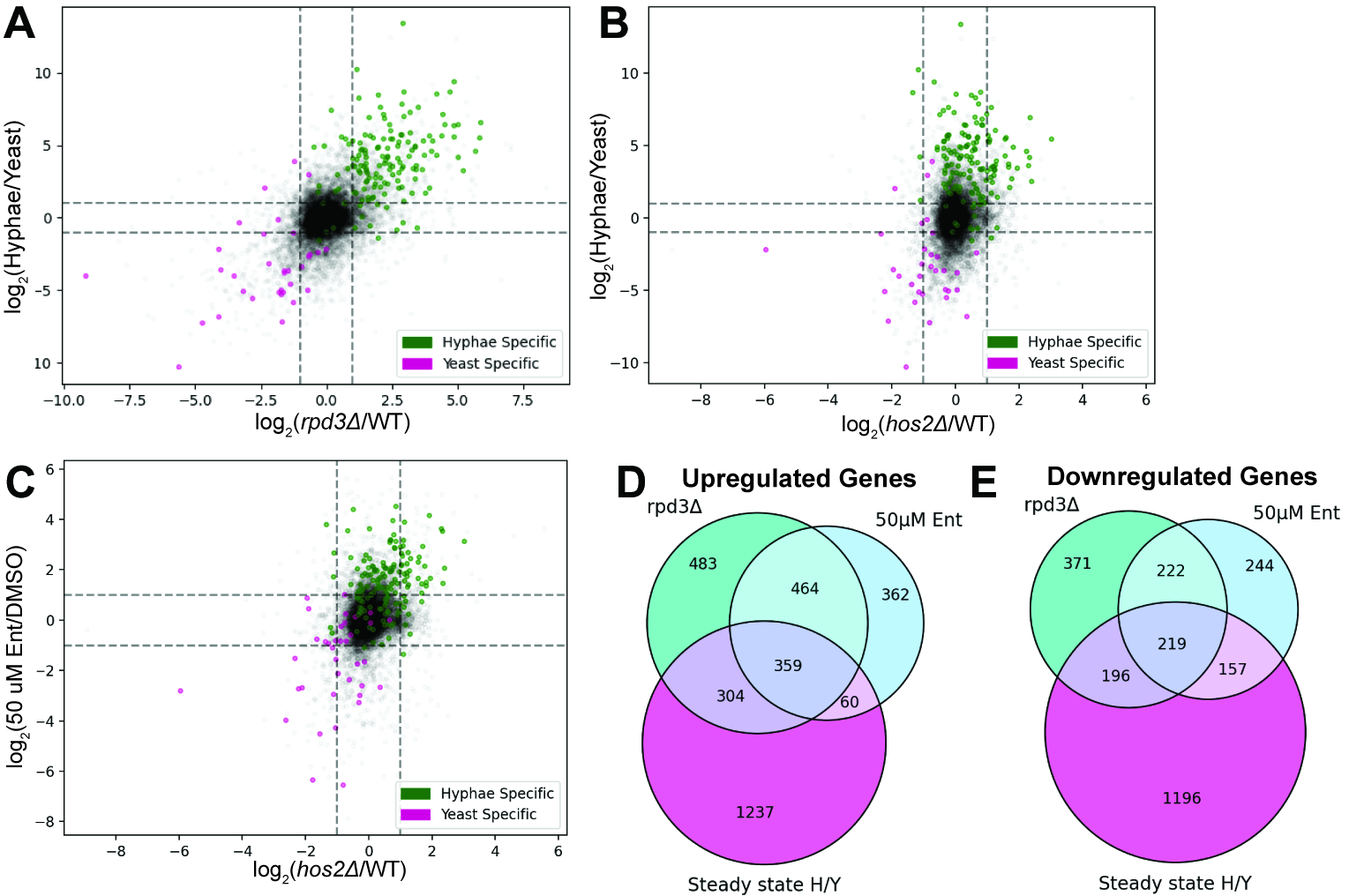

Supplement: S5 Fig — Scatterplots comparing differential RNA-seq signal in all G217B transcripts between steady-state hyphae and the following: rpd3Δ at 37 °C (A) and hos2Δ at 37 °C (B). Venn diagrams comparing the regulons identified in 2-day 50 µM Entinostat-treated cells, rpd3Δ at 37 °C, and steady-state hyphae (Gilmore and colleagues [24]) to identify shared and unique sets of upregulated (C) and downregulated (D) genes. E. Global scatterplot comparing differential RNA-seq signal as in (A, B) between 37 °C signal for hos2Δ cells and cells grown with 50 µM Entinostat for 2 days. Underlying data can be found in S6 and S7 Tables. (TIF) [file pbio.3003341.s005.tif]

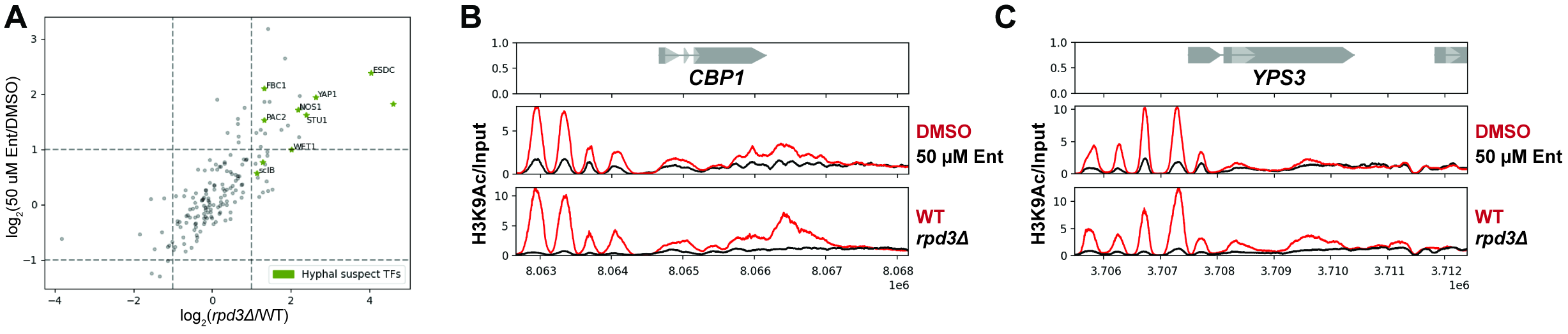

Supplement: S6 Fig — A. Scatter plot displayed in Fig 4F indexed on TF-encoding genes in the G217B genome. The green stars point out candidate hyphal TFs that are significantly induced under one of the two Rpd3-disrupting conditions. Underlying data can be found in S6 and S7 Tables. Coverage tracks displaying fold enrichment traces for H3K9Ac signal surrounding the loci of yeast-associated genes. Red traces indicate signal in control cells for both conditions in the loci surrounding CBP1 (B) and YPS3 (C). The underlying data for these panels can be found in GSE316185, GSE316355, and GSE316386. (TIF) [file pbio.3003341.s006.tif]

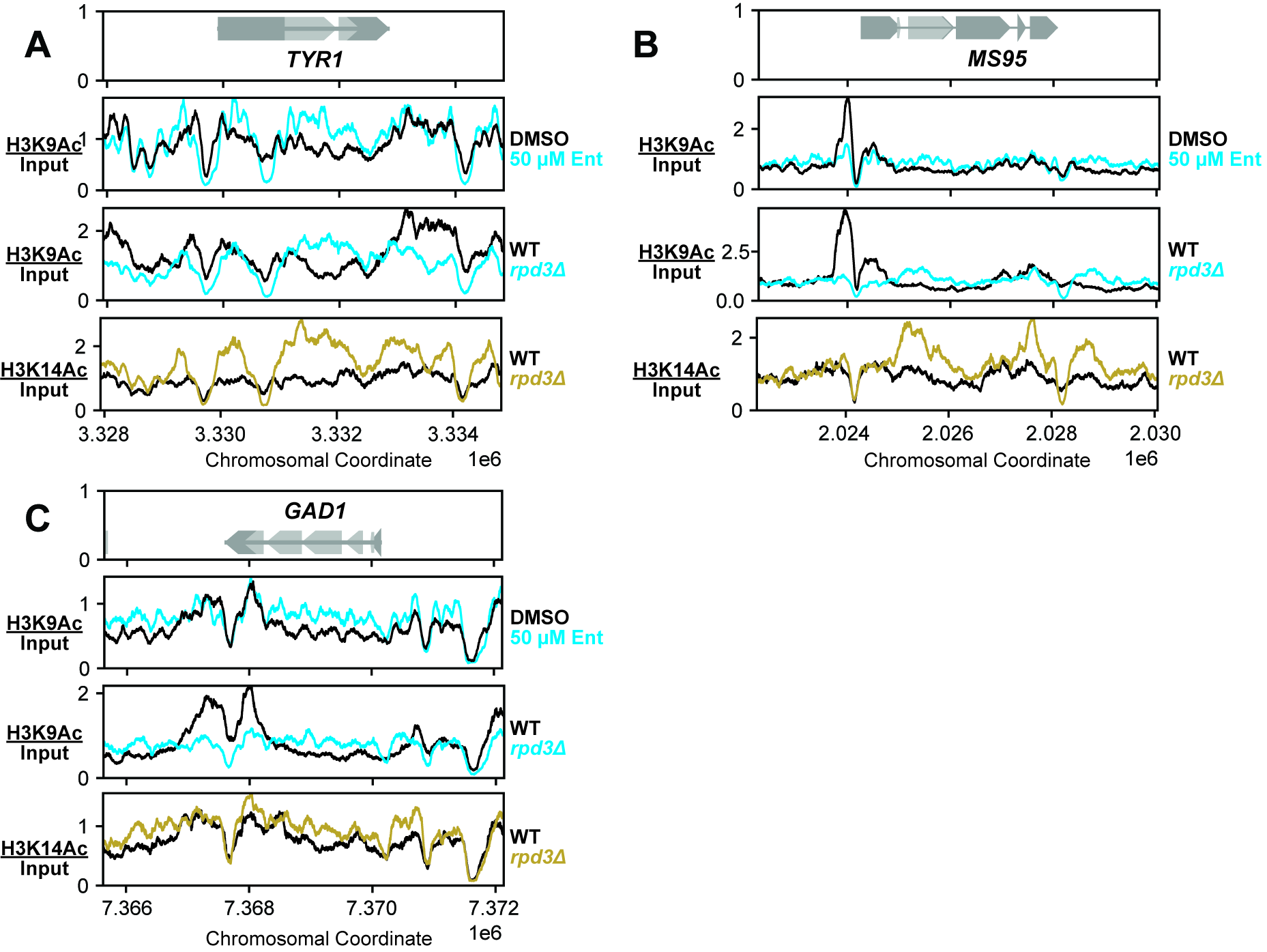

Supplement: S7 Fig — Coverage tracks displaying traces of genomic ChIP/input signal for pulldowns on two different histone H3 acetylation marks, H3K9Ac and H3K14Ac. 2-day 37 °C cultures of WT G217B yeast treated with DMSO or 50 µM Entinostat along with G217B WT and rpd3Δ strains were used for chromatin isolation and subsequent pulldown. Colored traces (H3K9Ac in red and H3K14Ac in green) depict the signal in WT or DMSO control cells while the black traces represent signal with Entinostat or in rpd3Δ surrounding the loci of TYR1 (A), GAD1 (B), and MS95 (C). The underlying data for these panels can be found in GSE316185, GSE316355, and GSE316386. (TIF) [file pbio.3003341.s007.tif]
