# Supplementary material for: The Rpd3 histone deacetylase is a critical regulator of temperature-mediated morphogenesis and virulence in the human fungal pathogen Histoplasma
Source: PLoS Biol. 2026 Mar 17;24(3):e3003341. doi: 10.1371/journal.pbio.3003341 (PMC13132440; doi:10.1371/journal.pbio.3003341)

Western Blots shown in Fig. 5B

Ryp1:

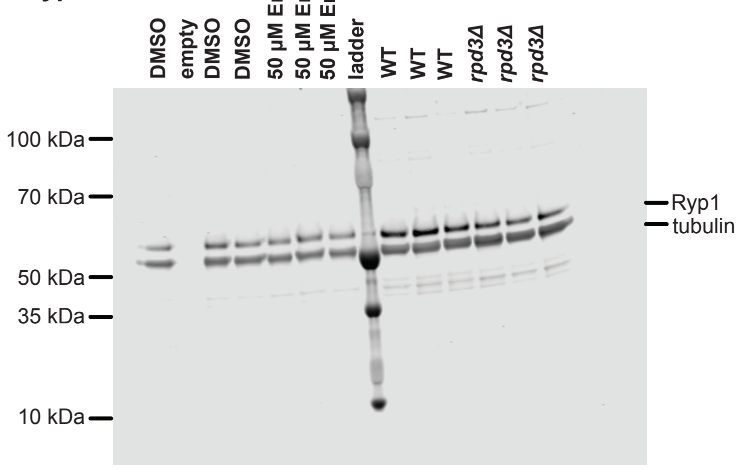

Ryp2:

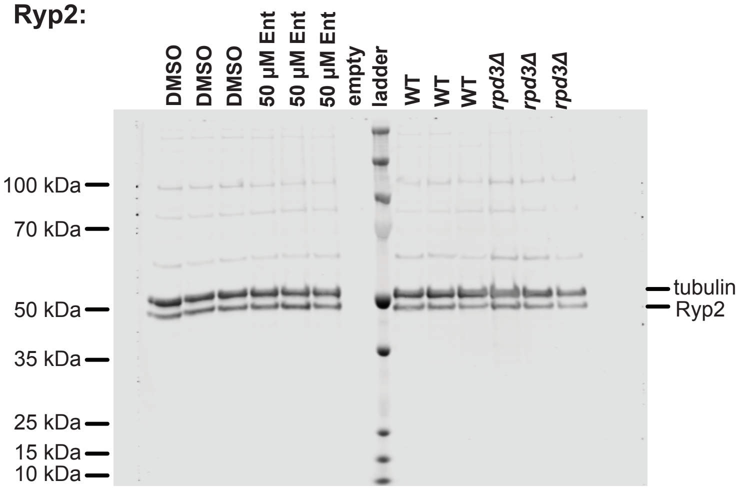

Ryp3 :

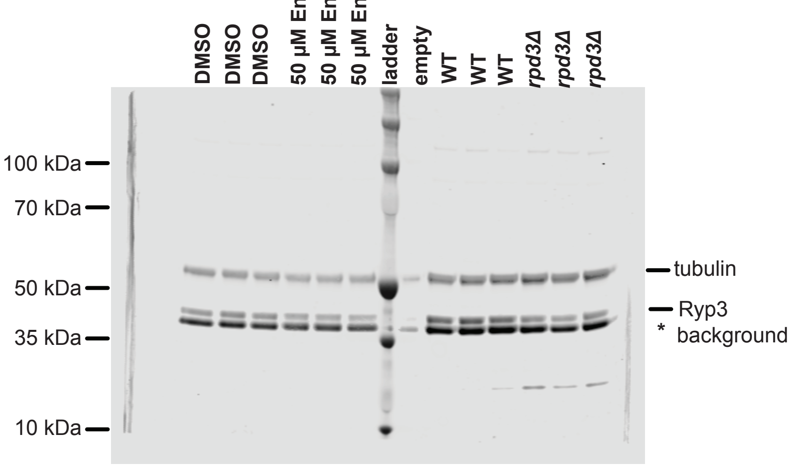

Coommassie shown in Fig. 7B

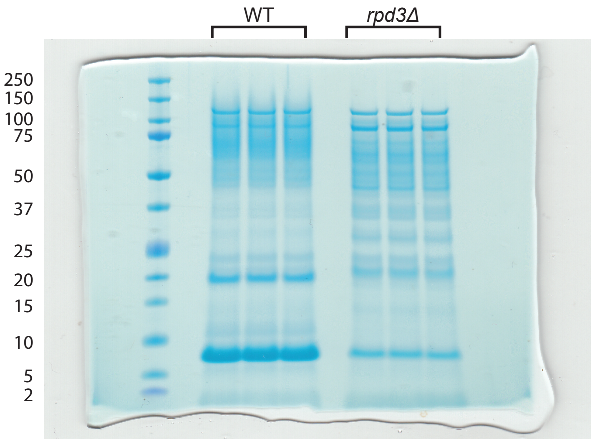

For all western blots: Protein samples from DMSO and 50 µM treated cells were obtained from samples used for ChIP studies in Figure 5. Protein samples from WT and *rpd3Δ* cells were obtained from samples used for 37C transcriptomics studies in Figure 4.

Agarose Gel shown in Fig. S3A

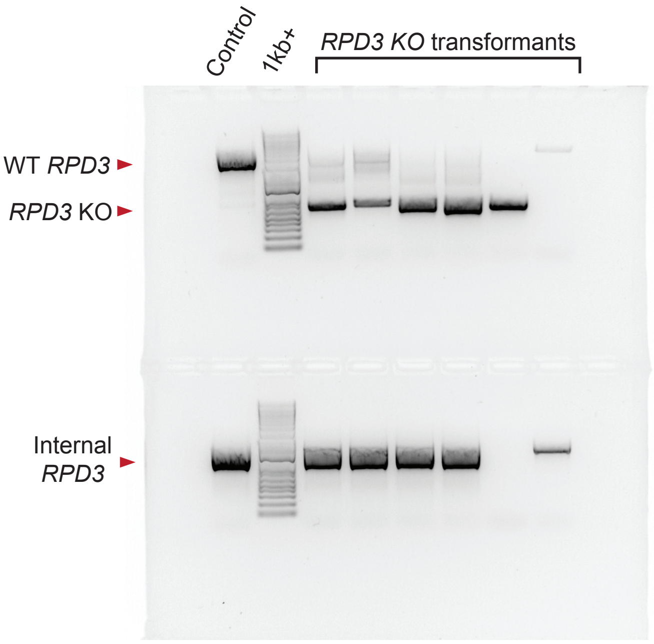

Agarose Gel shown in Fig. S3B

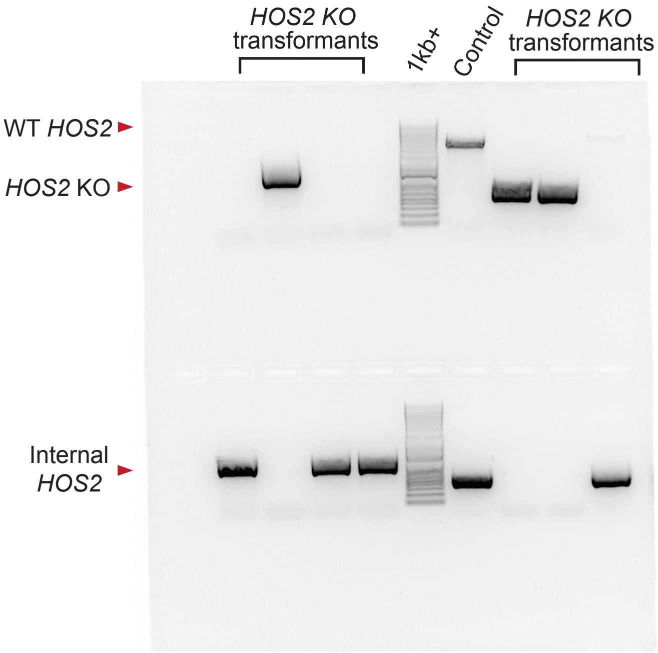

Supplement: S1 Raw Images — (PDF) [file pbio.3003341.s027.pdf]
